# The role of *Tal2* and *Tal1* in the differentiation of midbrain GABAergic neuron precursors

**DOI:** 10.1242/bio.20135041

**Published:** 2013-08-09

**Authors:** Kaia Achim, Paula Peltopuro, Laura Lahti, Hui-Hsin Tsai, Alyssa Zachariah, Mia Åstrand, Marjo Salminen, David Rowitch, Juha Partanen

**Affiliations:** 1 Department of Biosciences, P.O. Box 56, Viikinkaari 5, FIN00014-University of Helsinki, Helsinki, Finland; 2 Department of Pediatrics and Howard Hughes Medical Institute, University of California, San Francisco, 513 Parnassus Avenue, San Francisco, CA 94143-0734, USA; 3 Department of Veterinary Biosciences, P.O. Box 66, Agnes Sjobergin katu 2, FIN00014-University of Helsinki, Helsinki, Finland

**Keywords:** Neurogenesis, GABAergic neuron, Ventral tegmental area (VTA), Substantia nigra pars reticulata (SNpr), Midbrain, Dopaminergic neuron, Hindbrain, Rhombomere 1, Transcription factor, Gata, Tal, Scl, Brain development, Mouse

## Abstract

Midbrain- and hindbrain-derived GABAergic interneurons are critical for regulation of sleep, respiratory, sensory-motor and motivational processes, and they are implicated in human neurological disorders. However, the precise mechanisms that underlie generation of GABAergic neuron diversity in the midbrain–hindbrain region are poorly understood. Here, we show unique and overlapping requirements for the related bHLH proteins Tal1 and Tal2 in GABAergic neurogenesis in the midbrain. We show that Tal2 and Tal1 are specifically and sequentially activated during midbrain GABAergic neurogenesis. Similar to Gata2, a post-mitotic selector of the midbrain GABAergic neuron identity, Tal2 expression is activated very early during GABAergic neuron differentiation. Although the expression of Tal2 and Gata2 genes are independent of each other, Tal2 is important for normal midbrain GABAergic neurogenesis, possibly as a partner of Gata2. In the absence of Tal2, the majority of midbrain GABAergic neurons switch to a glutamatergic-like phenotype. In contrast, Tal1 expression is activated in a Gata2 and Tal2 dependent fashion in the more mature midbrain GABAergic neuron precursors, but Tal1 alone is not required for GABAergic neuron differentiation from the midbrain neuroepithelium. However, inactivation of both Tal2 and Tal1 in the developing midbrain suggests that the two factors co-operate to guide GABAergic neuron differentiation in a specific ventro-lateral midbrain domain. The observed similarities and differences between Tal1/Tal2 and Gata2 mutants suggest both co-operative and unique roles for these factors in determination of midbrain GABAergic neuron identities.

## Introduction

The mammalian midbrain and hindbrain contain neuronal populations that control motivation, motor and sensory function as well as vital autonomic activity and sleep.

Gamma-aminobutyric acid (GABA) is the primary inhibitory neurotransmitter in the mature brain and is used by hundreds of different types of neurons throughout the central nervous system (CNS). In the midbrain, distinct GABAergic precursors are generated in ventro-lateral and dorsal progenitor domains m1–m5 (Nakatani et al., 2007; Kala et al., 2009). These precursors are thought to contribute to diversified GABAergic neurons in the midbrain including superior and inferior colliculi, periaqueductal gray area and nuclei in the midbrain reticular formation (mRF), where they are involved in functions such as processing of sensory information (Tsunekawa et al., 2005; Kala et al., 2009; Peltopuro et al., 2010; Lahti et al., 2013).

Other GABAergic neurons associate with dopaminergic (DA) nuclei, ventral tegmental area (VTA) and substantia nigra (SN), in the ventral midbrain. These ventral midbrain GABAergic neurons (vMB GABAn) are critical for the activity of the DA pathways and regulation of voluntary movements. Furthermore, the vMB GABAn embedded in the VTA and rostromedial tegmental nucleus (RmTg; also called the “tail of VTA”) have recently been found to be central for regulation of motivational states and reward (Jhou et al., 2009; Kaufling et al., 2010; Hong et al., 2011; Cohen et al., 2012; Lahti et al., 2013). Thus, the vMB GABAn are critical for understanding of aetiology and potentially also treatment of neurological and psychiatric disease, such as Parkinson's disease, mood disorders, addiction and schizophrenia. In contrast to other midbrain GABAergic neurons, which are derived from the midbrain neuroepithelium, the vMB GABAn population originates from the rhombomere 1 (r1) neuroepithelium and migrates rostrally to the midbrain as post-mitotic young neuronal precursors (Achim et al., 2012). In addition, the GABAergic neurons in the diencephalic part of SN have a distinct origin, likely in the more anterior brain regions. Thus, the developmental history of the midbrain GABAergic neurons is complex and involves contributions from adjacent brain regions, especially the r1.

Underlying the segmental organization of diversified GABAergic neuron development are organizational signals in the dorsal–ventral and anterior–posterior axes that instruct expression of transcription factors in distinct and overlapping patterns (Puelles, 2007; Nakamura et al., 2008). In the midbrain and posterior diencephalon, a zinc-finger transcription factor (TF) *Gata2* is expressed upon cell cycle exit of all GABAergic precursors and controls their differentiation (Kala et al., 2009; Willett and Greene, 2011; Virolainen et al., 2012). Here *Gata2* appears to function as a terminal selector gene of the GABAergic neuron identity. Without Gata2, all the midbrain derived GABAergic precursors switch to a glutamatergic phenotype. In contrast to the midbrain, *Gata2* is not expressed in the anterior forebrain GABAergic precursors (Parras et al., 2002; Petryniak et al., 2007), and while it is expressed in the r1 it is functionally dispensable for GABAergic neurogenesis, possibly because of co-expression of *Gata3* (Kala et al., 2009; Achim et al., 2012). In addition to GABAergic neurons, *Gata2* and *Gata3* are also expressed in the r1 serotonergic neurons and Gata2 is required for their development (Craven et al., 2004; Kala et al., 2009).

Gata TFs can exert different and even opposing effects on the same targets and their functions are highly context-dependent (Wozniak et al., 2008). These differences can be explained by participation of other regulatory factors. Indeed, Gata factors typically function as a part of a multi-protein transcription regulatory complex that may include Friend of Gata (Fog) family proteins Fog1 and Fog2, LIM-only TF Lmo2, LIM-family cofactor Nli (Ldb1), bHLH TF Tal1 (Scl) and others (reviewed by Cantor and Orkin, 2002; Grosveld et al., 2005). In cultured blood cell progenitors, the presence or absence of Tal1 in the Gata2-complex can define the nature of target gene regulation (Tripic et al., 2009). The Tal1–Gata2 interaction has also been shown to regulate neuronal fate selection in the ventral spinal cord (Zhou et al., 2000; Karunaratne et al., 2002; Muroyama et al., 2005). Here, Tal1 appears to specifically instruct the development of V2b GABAergic neurons from an initially bipotent postmitotic precursor pool (Del Barrio et al., 2007; Peng et al., 2007). The precursor fate segregation also involves a cofactor Lmo4, which mediates the formation of Gata2–Tal1 transcriptional complex that turns on *Gata2/3* expression and other targets in V2b cells (Joshi et al., 2009).

Although their cell-type specificity has not been demonstrated, expression of *Tal1* and a related gene *Tal2* have been detected in the mouse midbrain and r1 raising the possibility of unique or overlapping interactions with Gata2 (Elefanty et al., 1999; Mori et al., 1999; Herberth et al., 2005). Furthermore, conditional deletion of *Tal1* in CNS precursors has been shown to result in a slight reduction of *Tal1* expressing neurons in the midbrain and more pronounced loss in the hindbrain (Bradley et al., 2006). Recently, we showed that *Tal1* is expressed and required in a ventral subdomain of the r1 GABAergic precursors (Achim et al., 2012). Consistent with their origin in the r1, the number of vMB GABAn are greatly reduced in the *Tal1* mutants. Also, Tal2 has been shown to be important for development of superior and inferior colliculi (Bucher et al., 2000), although precise cell type or stage specific roles have not been described.

Here, we established the functions of Tal1 and Tal2 in GABAergic neurogenesis in the developing midbrain using single and compound mutant mice. We show that *Tal1* and *Tal2*, together with *Gata2* and *Gata3*, are activated in spatially and temporally specific fashion in the differentiating GABAergic precursors, and that unique functions of these factors are necessary components of GABAergic sub-type specification in distinct regions of the developing brain.

## Materials and Methods

### Mice

The following mouse lines and their genotyping have been described previously: *En1^Cre^* (Kimmel et al., 2000) provided by Wolfgang Wurst, Helmholz Centre Munich, Germany; *Wnt1^Cre^* (Danielian et al., 1998) Jackson Laboratory, Bar Harbor, USA; *Gata2^flox^* (Haugas et al., 2010), *Tal1^flox^* (Bradley et al., 2006) provided by David Curtis Monash University, Melbourne, Australia and Stuart Orkin, Dana-Farber Cancer Institute, Boston, USA; *Tal2^null^* (Bucher et al., 2000) provided by Terry Rabbitts, MRC Weatheral Institute of Molecular Medicine, Oxford, UK; and *Gad67^GFP^* (Tamamaki et al., 2003) provided by and Yuchio Yanagawa, Gunma University Graduate School of Medicine, Maebashi, Japan. For staging, the day of vaginal plug was counted as embryonic day 0.5 (E0.5). For *in situ* mRNA hybridization (ISH) and immunohistochemistry (IHC) embryos and brains were fixed in 4% paraformaldehyde in 1× PBS at RT for 2–7 days. Samples were dehydrated, embedded in paraffin (Merck), sectioned at 5 µm, and collected on adjacent slides. All analyses were confirmed using 2–5 biological replicates (*e.g.* mutant embryos from different litters of same stage). All experiments were approved by the Laboratory Animal Center of the University of Helsinki, Finland and the Institutional Animal Care and Use Committee of the University of California, San Francisco.

### In situ mRNA hybridization and immunohistochemistry

mRNA ISH analyses on paraffin sections were performed as described previously (Wilkinson and Green, 1990) using ^35^S- or digoxigenin-labeled cRNA probes. IHC was performed as described previously (Kala et al., 2008). For combined ISH and IHC, TSA Fluorescence Palette System (PerkinElmer) was used to visualize ISH signal. Additional primary antibodies were added after the ISH signal detection. For defining of the domain boundaries in the midbrain (m3–m5), adjacent sections were analysed for Helt, Nkx2.2 and Nkx6.1 expression. Detailed ISH and IHC protocols are available upon request.

Mouse cDNA probes used for ISH analysis were: *Gad1 (Gad67)*, *Slc17a6 (Vglut2)* (Guimera et al., 2006), *Gata2*, *Gata3* (Lilleväli et al., 2004), *Six3* (IMAGE 761326), *Tal1* (IMAGE 6826611) *Tal1flox* (detects specifically the *Tal1* transcript from the wild-type or unrecombined locus; contains the sequences between the LoxP sites in the *Tal1^flox^* allele (nt 699–1104 of NM_011527)), *Tal2* (IMAGE 40051579). Following antibodies were used in IHC stainings: rabbit anti-Gata2 (Santa Cruz sc-9008, 1:250), mouse anti-Gata3 (Santa Cruz sc-268, 1:200), goat anti-GFP (Abcam ab6673, 1:500), rabbit anti-GFP (Abcam ab290, 1:500), guinea pig anti-Heslike (Helt, 1:500; gift from Ryoichiro Kageyama, Institute for Virus Research, Kyoto University, Japan), mouse anti-HuC/D (Molecular Probes A21271, 1:1000), goat anti-Otx2 (R&D Systems BAF1979, 1:200), mouse anti-Ascl1 (BD Biosciences 556604, 1:200), mouse anti-Nkx2-2 (DSHB 74.5A5, 1:250), mouse anti-Nkx6.1 (1:1000, DSHB F55A10), mouse anti-Sox2 (Millipore AB5603, 1:400), mouse anti-TH (Millipore MAB318, 1:800), rabbit anti-5-HT (Immunostar 20080, 1:1500). Alexa-488 and Alexa-568 conjugated goat anti-rabbit IgG and anti-mouse IgG, donkey anti-rabbit IgG, anti-mouse IgG and anti-goat IgG (1:400, Invitrogen) were used as secondary antibodies.

### Microscopy

IHC and ISH staining on paraffin sections were visualized with an Olympus AX70 microscope with Olympus DP70 camera or a Zeiss Axioimager M2 microscope with Axiocam HRc camera. Images were processed and assembled with Adobe Photoshop software. Confocal images were taken as snapshots with Leica SP5 confocal microscope.

## Results

### *Tal1* and *Tal2* are co-expressed with Gata2 in developing GABAergic neurons of the midbrain and r1

**Fig. 1.**
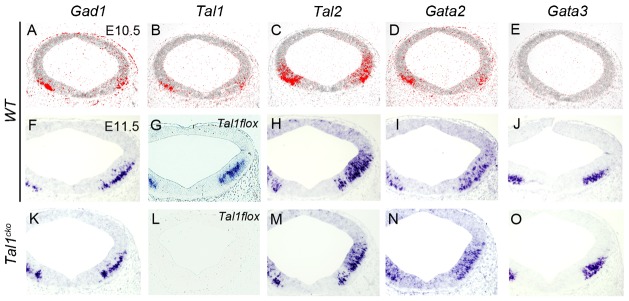
Expression of Tal and Gata factors in the wild-type and *Tal1^cko^* midbrain. ISH analysis of *Gad1*, *Tal1*, *Tal2*, *Gata2*, and *Gata3* expression on adjacent coronal sections of *WT* (**A–J**) and *Tal1^cko^* (**K–O**) midbrains at E10.5–E11.5. Radioactive ISH in panels A–E, non-radioactive in the others. The development of GABAergic neurons remains unaffected in the *Tal1^cko^* midbrain. *Tal1flox* probe (G,L) was designed to recognize the region between LoxP sites in the conditional allele and therefore the loss of signal in the *Tal1^cko^* midbrain demonstrates efficient inactivation of the *Tal1* gene.

**Fig. 2.**
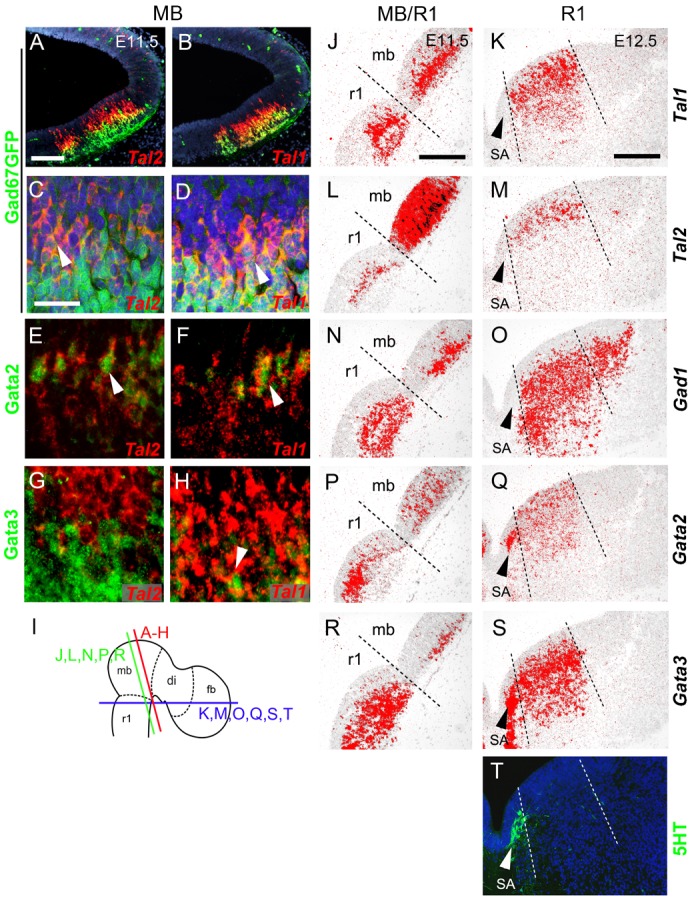
Expression of Tal and Gata factors in the developing midbrain and r1. (**A–H**) Co-expression of *Tal2* and *Tal1* (ISH) with Gad67-driven GFP, Gata2, and Gata3 in coronal sections of E11.5 midbrains. Epifluorescence images in (A,B,E–H), confocal images in (C,D). (**I**) The planes of sectioning. (**J–S**) Radioactive ISH with the probes indicated adjacent coronal sections from E11.5 midbrain–r1 (J,L,N,P,R). The dashed line indicates the midbrain–r1 border, defined by the anti-Otx2 staining on an adjacent section (not shown). (K,M,O,Q,S) Radioactive ISH with the probes indicated on parallel transverse sections from E12.5 rhombomere 1 area. The dashed lines mark the ventral and dorsal borders of *Nkx6.1* expression domain in r1 (not shown). The serotonergic domain (SA) was detected using with an anti-5HT antibody on a parallel section (**T**). r1, rhombomere 1, mb, midbrain. White arrowheads in C–H indicate co-expression, arrowheads in K–T point to serotonergic neurons. Scale bars: 100 µm (A,J), 25 µm (C), 200 µm (K).

**Fig. 3.**
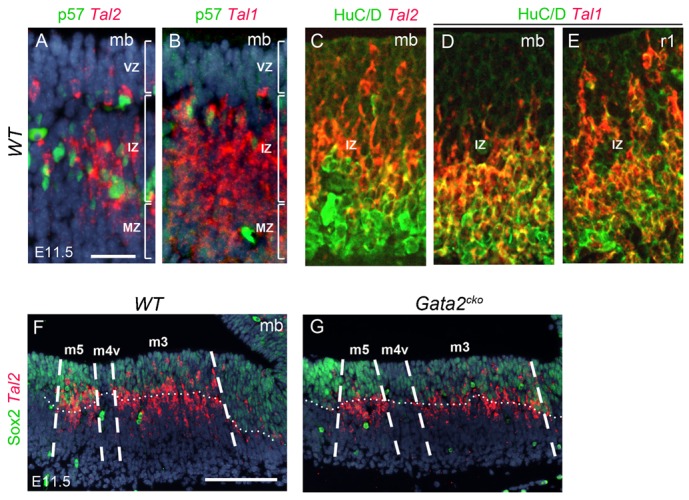
Expression of Tal factors in the intermediate and mantle zones of the midbrain. (**A**,**B**) Fluorescent ISH with *Tal1* and *Tal2* probes combined with p57 IHC on E11.5 ventral midbrain. The positions of three zones were deduced from the p57+ cells, which mark the intermediate zone. (**C–E**) Fluorescent ISH with *Tal1* and *Tal2*, and IHC with a pan-neuronal marker HuC/D on E11.5 ventral midbrain. (**F**,**G**) Fluorescent ISH with *Tal2* and IHC with ventricular zone marker Sox2 on E11.5 wild-type and *Gata2^cko^* ventral midbrain, with domain borders indicated. The dashed line visualizes the border between ventricular zone and mantle zone, based on Sox2 staining. VZ, ventricular zone; IZ, intermediate zone; MZ, mantle zone; mb, midbrain; r1, rhombomere 1. Scale bars: 25 µm (A–E), 100 µm (F,G).

We first analysed the expression of *Tal* genes in the wild-type midbrain and r1 regions by ISH during GABAergic neurogenesis at E10.5–E12.5. In the midbrain, we detected *Tal2* expression in the ventricular zone (VZ) and intermediate zone (IZ; Fig. 1C,H, Fig. 2A, Fig. 3A,C,F), while *Tal1* was confined to the IZ and mantle zone (MZ; Fig. 1B,G, Fig. 2B, Fig. 3B,D). In addition, both *Tal1* and *Tal2* were expressed in the GFP^+^ GABAergic neurons in the *Gad67^GFP/+^* mouse embryos (Fig. 2A–D). In contrast to the midbrain, *Tal2* expression was very low and restricted to a small IZ area in the anterior r1 (Fig. 2L,M) while *Tal1* expression in r1 was robust and extended from the VZ to MZ (Fig. 2J,K, Fig. 3E).

In both midbrain and r1, expression domains of *Tal1/2* and *Gata2/3* appeared to overlap extensively (Fig. 1, Fig. 2J–S), with the exception of Gata2/3^+^ serotonergic neuron precursors in the r1 (Fig. 2K,M,O,Q,S,T). To further study the co-expression of *Tal1/2* and *Gata2/3*, we combined ISH for *Tal1/2* and IHC for Gata2/3. This showed that in the midbrain, Gata2 is nearly exclusively co-expressed with *Tal2* in the VZ and IZ (Fig. 2E). *Tal1* is activated later during differentiation, and thus Gata2 and *Tal1* are co-expressed in the IZ (Fig. 2F). *Tal1* positive cells in the MZ most abundantly expressed Gata3 (Fig. 2H). In contrast, we detected only little co-expression of *Tal2* and Gata3 in the MZ (Fig. 2G). Both *Tal1* and *Tal2* were detected in postmitotic (HuC/D+) neurons (Fig. 3C,D). In the r1, the weak expression of *Tal2* precluded fluorescent ISH-based co-localization studies. However, radioactive ISH demonstrated that *Tal2* expression was mostly confined to the IZ in the r1 (Fig. 2M). In contrast to the midbrain, *Tal1* expression was detected already in the VZ and early during post-mitotic differentiation of the r1 GABAergic neurons (Fig. 2K, Fig. 3E). In summary, *Tal1* and *Tal2* specifically mark the midbrain and r1 GABAergic precursors and co-localize with *Gata2* and *Gata3*. However, the dynamics of *Tal1* and *Tal2* expression differ between the midbrain and r1. While *Tal2* expression precedes *Tal1* in the midbrain, the opposite is observed in the r1 where *Tal1* is expressed at an earlier stage and co-expressed with Gata2 already in the VZ.

### Gata2 is required for the expression of *Tal1* but not *Tal2* in the midbrain

**Fig. 4.**
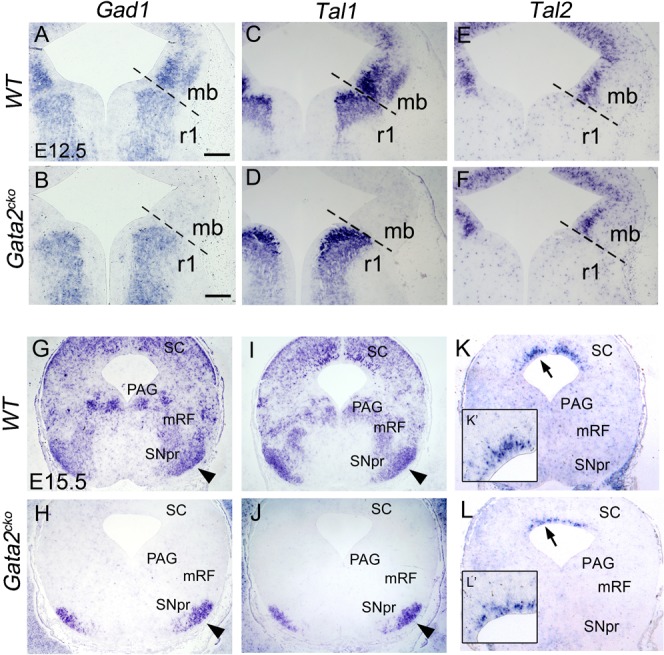
*Tal1* and *Tal2* expression in the *Gata2* mutants. ISH analysis of *Gad1* (**A**,**B**,**G**,**H**), *Tal1* (**C**,**D**,**I**,**J**) and *Tal2* (**E**,**F**,**K**,**L**) expression in wild-type *(WT)* and *Gata2^cko^* embryos at E12.5 and at E15.5. Note that the expression of *Tal2* in the r1 is below the detection level by these non-radioactive ISH experiments. The dashed line indicates the midbrain/r1 border, detected by Otx2 staining on a parallel section (data not shown). Arrowheads point to the SNpr, defined by TH immunostaining on adjacent sections (not shown). Arrows point to *Tal2* expression domain in the dorsal midbrain (close up images in insets, **K′** and **L′**). mb, midbrain; r1, rhombomere 1; PAG, periaqueductal grey; mRF, midbrain reticular formation; SNpr, substantia nigra pars reticulata, SC, superior colliculi. Scale bars: 200 µm.

To study if activation of *Tal1* and/or *Tal2* requires *Gata2* function, we analysed *En1^Cre^*; *Gata2^flox/flox^* (*Gata2^cko^*) mutants in which both midbrain and r1 are devoid of Gata2 expression (Kala et al., 2009). In the *Gata2^cko^* midbrain, *Tal1* was downregulated at E12.5 (Fig. 4C,D) together with GABAergic neuron markers *Gad1*, *Gata3* and *Six3* (Fig. 4A,B and data not shown). In contrast, we detected persistent *Tal2* mRNA expression (Fig. 4E,F, Fig. 3G). In the r1, expression of *Gad1* and *Tal1/2* was not affected by the loss of *Gata2* function (Fig. 4B,D,F and data not shown), indicating that Gata2 is required for *Tal1* expression in the midbrain but not in the r1, while *Tal2* expression is Gata2-independent, at least in the midbrain.

We also analysed *Gad1*, *Tal1* and *Tal2* expression in wild-type and *Gata2^cko^* midbrains at E15.5 (Fig. 4G–L). Consistent with expression of *Tal1* in the MZ at E12.5, *Tal1* was abundantly expressed in the wild-type midbrain regions containing GABAergic neurons. In contrast, the expression of *Tal2* was restricted to the VZ/IZ of the most dorsal midbrain, where GABAergic neurogenesis continues later than in the ventro-lateral regions (Achim et al., 2012). In the *Gata2^cko^* midbrain, *Tal1* expression was abolished, except for the r1-derived GABAergic neurons in the SNpr (Fig. 4I,J). As in the ventro-lateral midbrain at E12.5, *Tal2* was still expressed in the dorsal midbrain of *Gata2^cko^* mutants at E15.5, although its pattern of expression was more narrow suggesting more rapid downregulation in the IZ (Fig. 4K,K′,L,L′).

### Tal factors are required to activate genes characteristic for differentiating GABAergic neurons in the midbrain

To elucidate the importance of different Tal factors in regulation of GABAergic neuron differentiation in the midbrain and r1, we studied the expression of several genes characteristic to midbrain and r1 GABAergic neurons (*Gata2*, *Gata3*, *Tal1*, *Six3*, *Gad1*) in embryos mutant for *Tal2* (*Tal2^null/null^* = *Tal2^ko^*), *Tal1* (*En1^cre/+^*; *Tal1^flox/flox^* = *Tal1^cko^*), or both *Tal1* and *Tal2* (*Wnt1^cre/+^*; *Tal1^flox/flox^*; *Tal2^null/null^* = *Tal1/2^dko^*) in comparison with the *Gata2^cko^* mutants. *Wnt1^Cre^* was used for inactivation in *Tal1/2^dko^* double mutants due to availability of the mouse strains. In the *Tal1^cko^* mutants, *En1^cre^*-mediated inactivation of *Tal1^flox^* allele occurs throughout the midbrain and r1. In the *Tal1/2^dko^*, the *Tal1^flox^* allele is inactivated by *Wnt1^cre^*, which is active mostly in the midbrain. Both *Wnt1^cre^*- and *En1^cre^*-mediated recombination is complete by E8.5, well before the onset of neuronal differentiation (Trokovic et al., 2003). We detected no *Tal1* expression in the midbrain and r1 of the *Tal1^cko^* or *Tal1/2^dko^* embryos at E11.5–E12.5 (Fig. 1L and data not shown). At E11.5, inactivation of *Tal1* alone (*Tal1^cko^*) did not affect GABAergic neurogenesis or the expression of *Gata2*, *Gata3* or *Tal2* in the midbrain (Fig. 1K–O).

**Fig. 5.**
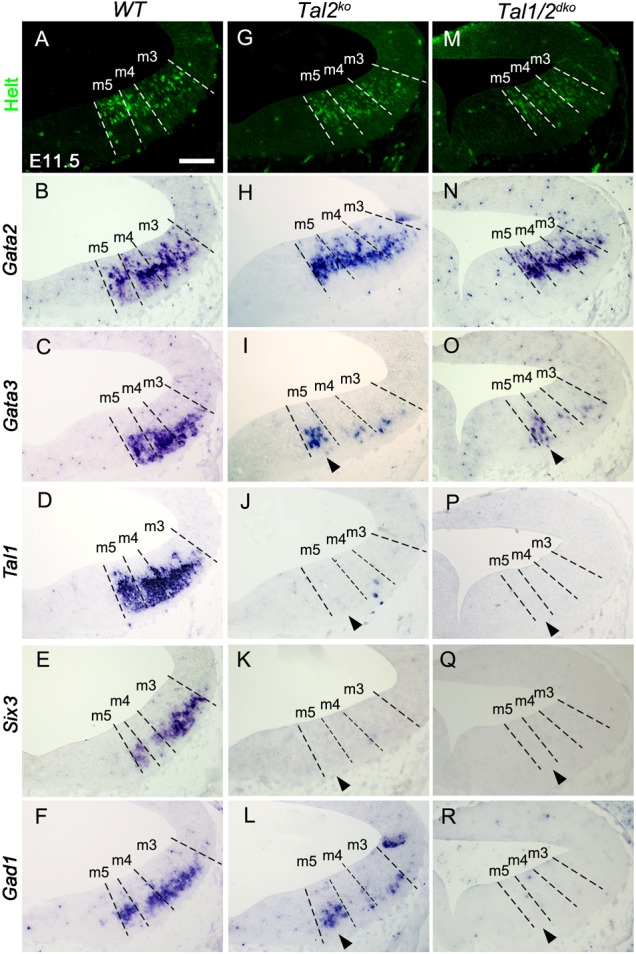
GABAergic gene expression in *Tal2* and *Tal1/2^dko^* mutant midbrains. Expression of Helt (IHC), *Gata2*, *Gata3*, *Tal1*, *Six3* and *Gad1* (ISH) on coronal sections of E11.5 wild-type (**A–F**), *Tal2^ko^* (**G–L**) and *Tal1/2^dko^* (**M–R**) midbrains. The dashed lines indicate the borders of midbrain m3–m5 domains, defined by Helt and Nkx2.2 stainings on parallel sections (not shown). The arrowheads point to the m5 domain, where fate transformation appears to be incomplete. Scale bar: 100 µm.

The VZ progenitors in the midbrain GABAergic regions appeared unaffected also in the *Tal2^ko^* (supplementary material Fig. S1) and *Gata2* expression was maintained in the early postmitotic precursors at E11.5 (Fig. 5H). In contrast, other post-mitotic GABAergic precursor specific genes were largely downregulated, in *Tal2^ko^* embryos (Fig. 5I–L). However, in contrast to *Gata2^cko^*, some *Gad1^+^* and *Gata3^+^* cells were still detected in the *Tal2^ko^* embryos, especially in the m5 domain (Fig. 5I,L, arrowheads).

Although *Tal1* expression was downregulated in the *Tal2^ko^* brains at E11.5 (Fig. 5J), and *Tal1* inactivation alone did not affect GABAergic neurogenesis in the midbrain (Fig. 1K–O) (Achim et al., 2012), it is possible that its expression at an earlier stage or at an undetectable level accounts for the survival of the few GABAergic cells observed in the *Tal2^ko^* mutants. To address this possible redundancy, we analyzed the *Tal1/2^dko^* double mutant midbrains. In these mutants, *Gad1* expression was lost completely in E11.5 midbrain (Fig. 5R), along with the GABAergic markers *Six3* and *Sox14* (Fig. 5Q and data not shown). However, we still detected *Gata3* expressing cells in the m5 domain of the *Tal1/2^dko^* midbrains (Fig. 5O), suggesting that the loss of GABAergic identity was still incomplete at least in this domain. Importantly, *Gata2* expression was unaffected in the *Tal1/2^dko^* (Fig. 3N), and the early GABAergic markers Helt (Fig. 5M) and Ascl1 (supplementary material Fig. S1) were detected in the VZ, confirming that the absence of Tal factors results in alterations only at the postmitotic differentiation stage.

In summary, our results suggest that *Tal2* and *Gata2* do not require each other for their expression but are independently activated in the midbrain and both control genes required for complete GABAergic differentiation and for the acquisition of correct neuronal identity. In addition, whereas Gata2 is required for GABAergic neurogenesis in the entire midbrain, requirement for the Tal factors for GABAergic development varies between the midbrain subdomains.

### Ectopic activation of glutamatergic neuron markers in *Tal* mutant midbrain

**Fig. 6.**
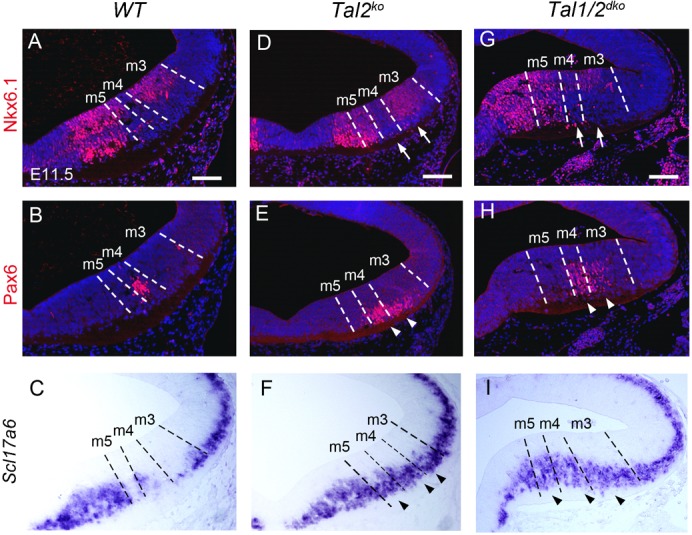
Glutamatergic gene expression in *Tal2* and *Tal1/2^dko^* mutant midbrains. Expression of Nkx6.1 (**A**,**D**,**G**), Pax6 (**B**,**E**,**H**) and Scl17a6 (**C**,**F**,**I**) on coronal sections of E11.5 wild-type (A–C), *Tal2^ko^* (D–F) and *Tal1/2^dko^* (G–I) midbrains. The dashed lines indicating the borders of m3–m5 domains were defined by Helt and Nkx2.2 stainings on parallel sections (not shown). Arrowheads point to ectopic expression. Arrows in panels D and G indicate areas where ectopic Pax6 expression is seen on a parallel section. Scale bars: 100 µm.

Concomitant to loss of GABAergic markers, we observed ectopic upregulation of glutamatergic neuron markers Pax6 and *Slc17a6* (Fig. 6E,F). These results indicate a GABAergic-to-glutamatergic fate transformation specifically in the midbrain of *Tal2^ko^* mutants, similar to the *Gata2^cko^* phenotype in the midbrain (Kala et al., 2009). However, unlike in *Gata2^cko^* mutants where only the m4v domain transforms to Pax6 expressing glutamatergic neurons whereas the m3 domain starts expressing m1–m2 glutamatergic marker Pou4f1 (Kala et al., 2009), in *Tal2^ko^* mutants ectopic Pax6 positive cells can be detected extensively in both m4d and m3 domains (Fig. 6E, arrowheads). Similar to the *Tal2^ko^* mutants, Pax6 and *Slc17a6* expression was upregulated in *Tal1/2^dko^* midbrain compared to wild type (Fig. 6H,I). In the wild type, Nkx6.1 is expressed in the VZ of domains m6, m5 and m3 and in the glutamatergic postmitotic precursors in m6. Nkx6.1 did not display ectopic expression in postmitotic Pax6 positive precursors derived from m4 or m3 in either *Tal2^ko^* or *Tal1/2^dko^* midbrains (Fig. 6A,D,G, arrows). However, especially in the *Tal1/2^dko^* mutants, Nkx6.1 expression appeared somewhat expanded in the postmitotic precursors in the m5 domain, similar to what has been observed in *Gata2^cko^* (Kala et al., 2009).

Thus, the Tal factors, and especially Tal2, appear to operate as post-mitotic selectors between GABAergic and glutamatergic neuronal phenotypes in the midbrain. In this respect, they share the function of their putative cofactor Gata2. However, Tal and Gata factors also differ in the ways they affect development of neuronal precursors derived from distinct midbrain regions.

### Loss of *Tal2* and *Gata2* results in changes in neuronal identities in the nuclei of perinatal midbrain

**Fig. 7.**
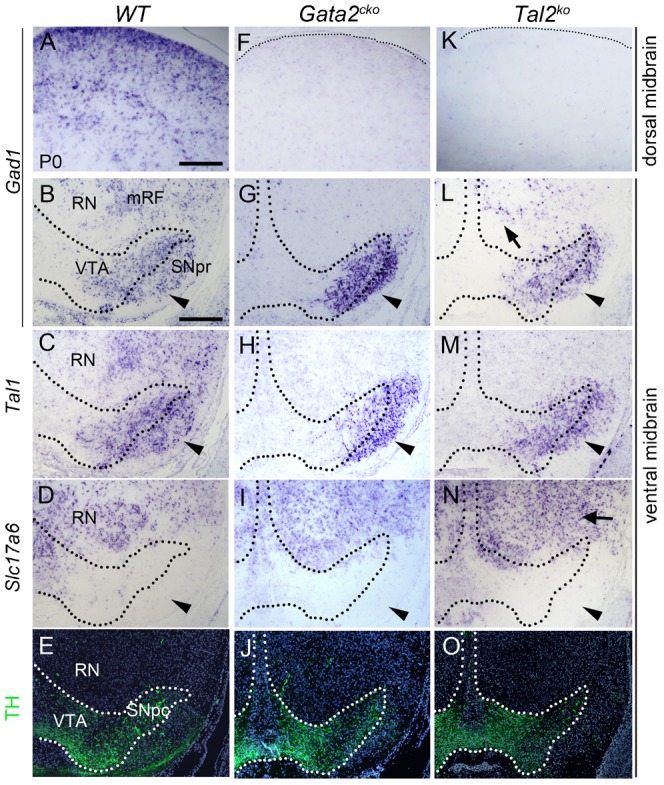
GABAergic and glutamatergic marker gene expression in the *Gata2^cko^* and *Tal2^ko^* perinatal brain. The expression of *Gad1*, *Tal1* and *Slc17a6* (ISH) was analyzed on coronal sections through the WT (**A–D**), *Gata2^cko^* (**F–I**), and *Tal2^ko^* (**K–N**) midbrains at P0. TH IHC on adjacent sections (**E**,**J**,**O**) was used as a landmark to identify the midbrain region and define the SN/VTA nuclei. The arrowheads point to SN, the arrow to mRF. Scale bars: 200 µm.

Next, we compared the expression of GABAergic and glutamatergic neuron markers in the midbrains of *Gata2^cko^* and *Tal2^ko^* mutants at perinatal stages. Normally, both *Gad1*^+^ GABAergic neurons and *Slc17a6*^+^ glutamatergic neurons are found in a variety of nuclei across midbrain (Fig. 7A,B,D). At E15.5–P0, *Gad1^+^* neurons co-expressed *Gata3* and *Tal1* in specific nuclei and midbrain regions including the SN (Fig. 4G,I, Fig. 7B,C and data not shown). In both *Gata2^cko^* and *Tal2^ko^* animals, GABAergic markers were completely lost in the dorsal midbrain including the superior colliculi (Fig. 7A,F,K and data not shown). Similarly, we found that *Tal2^ko^* greatly resembles *Gata2^cko^* in that the expression of GABAergic markers *Gad1* (Fig. 7B,G,L), *Gata3* and *Tal1* (Fig. 7C,H,M and data not shown) was lost (*Gata2^cko^*) or reduced (*Tal2^ko^*) in the mRF. Furthermore, in the SN/VTA marked by TH staining (Fig. 7E,J,O), *Gad1*, *Gata3* and *Tal1* expressing cells were retained both in the *Gata2^cko^* and *Tal2^ko^* (Fig. 7B,C,G,H,L,M, arrowheads, and data not shown). Consistent with cell fate re-specification, upregulation of *Slc17a6* was observed in the mRF region but not in SN/VTA of *Gata2^cko^* and *Tal2^ko^* midbrains (Fig. 7D,I,N). Similar changes were seen in *Tal1/2^dko^* (supplementary material Figs S2, S3; note for example the glutamatergic red nucleus (RN) which is surrounded by mostly GABAergic neurons in the wild type, but glutamatergic neurons in the mutants in supplementary material Fig. S3B,C,E,F). Thus, the perinatal *Tal2* and *Gata2* mutants display neuronal fate changes, which are consistent with their embryonic phenotypes. Furthermore, in contrast to *Tal1* (Achim et al., 2012), *Tal2* or *Gata2* are not required by the majority of vMB GABAn.

## Discussion

**Fig. 8.**
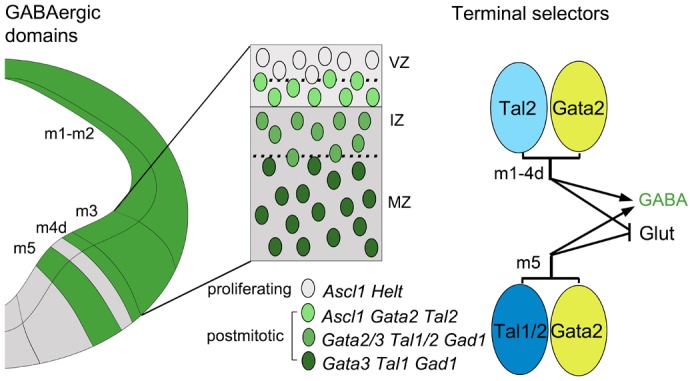
Model of Gata/Tal terminal selector complex and its variants in the midbrain. A schematic cross-section of E12.5 midbrain, with the domains generating GABAergic neurons indicated in green. Both *Gata2* and *Tal2* expression is initiated after cell cycle exit. Coincident expression of *Gata2* and *Tal2* leads to formation of a terminal selector complex crucial for the correct GABAergic identity development in m1–4d. In m5, Tal1 is also required. VZ, ventricular zone; IZ, intermediate zone; MZ, mantle zone; SA, serotonergic domain.

Knowledge of developmental regulatory mechanisms can provide key insights into generation of neuronal diversity and functional specialization in the CNS. Such mechanisms might also be involved in developmental anomalies leading to life-threatening human neurological disorders. For example, mutation of transcription factor PTF1A in humans results in abnormal development of GABAergic neurons in the dorsal r1 and cerebellar agenesis (Sellick et al., 2004; Hoshino et al., 2005). Development of GABAergic neurons is regulated by different molecular mechanisms in different parts of the developing CNS. Our previous studies identified Gata2 as a post-mitotic terminal selector gene during GABAergic neurogenesis in the embryonic midbrain and revealed developmental diversity of the distinct GABAergic neuron groups in the midbrain–r1 region (Kala et al., 2009; Achim et al., 2012). Here, we studied how the putative cofactors of Gata2, Tal1 and Tal2, are involved in the GABAergic neuron differentiation in the midbrain. Our results suggest that Tal2 is important for selection of GABAergic over glutamatergic neuron identity in the midbrain. In contrast, Tal1 marks more mature midbrain GABAergic precursors but is not required for their differentiation. Unlike Gata2, the requirement for Tal factors varies between the midbrain subregions. The ventro-lateral m5 domain appears the most tolerant to *Tal2* inactivation. Here, Tal1 and Tal2 may function redundantly to regulate GABAergic neurogenesis (Fig. 8).

### Similarities and differences of Gata and Tal factors as the terminal selectors of GABAergic identity

Studies on neuronal differentiation in various models, including *C. elegans*, have led to the concept of terminal selector genes (Hobert, 2011). These genes are thought to be active in post-mitotic neuronal precursors and regulate expression of gene batteries providing a neuron its identity. An important feature of a terminal selector gene is that its inactivation does not lead to the loss of the neuron but rather the loss, or transformation, of its identity. Our previous studies have suggested that *Gata2* fulfils most of the criteria of a terminal selector gene both in the developing midbrain and diencephalon (Kala et al., 2009; Virolainen et al., 2012). The results presented here suggest that the selection of the GABAergic identity also requires Tal2 in the developing midbrain. As Gata and Tal factors are involved in the same transcriptional complexes in other tissues, they may also form an analogous TF complex in the midbrain. Our results suggest that expression of both *Gata2* and *Tal2* is initiated very early after cell cycle exit and further demonstrate independent initiation of *Tal2* and *Gata2* expression, likely triggered by region- and cell-cycle stage specific cues. However, neither the endogenous Tal2 nor Gata2 alone are sufficient to induce all GABAergic neuron specific genes in the embryonic midbrain. Instead, we propose that the coincident expression of these two TFs leads to formation of a terminal selector complex of the GABAergic identity (Fig. 8).

Consistent with the hypothesis above, the midbrain phenotypes of *Gata2* and *Tal2* mutants are similar. In both cases progenitor proliferation, cell cycle exit and production of neuronal precursors appeared undisturbed, but the postmitotic precursors lost their GABAergic identity and acquired glutamategic characteristics instead. However, there also are some notable differences. In contrast to *Gata2* mutants, the GABAergic marker genes are not completely lost in the *Tal2* mutants. In particular, the m5 domain still retains some of its GABAergic characteristics without Tal2. Studies of the *Tal1/2^dko^* mutants suggest that this is partly explained by redundancy between Tal2 and Tal1. However, some GABAergic markers, such as *Gata3*, were detected in m5 even in the absence of both *Tal1* and *Tal2*. In the m3 domain, pronounced GABAergic-to-glutamatergic phenotypic transformation occurs in both *Tal2* and *Gata2* mutants. However, the ectopic glutamatergic neurons appear to be of different subtype in the two mutants. Without Gata2, the precursors produced in m3 upregulate *Pou4f1*, resembling the glutamatergic neurons born in m1 and m2 (Kala et al., 2009). In contrast, in the *Tal2* mutants, the ectopic glutamatergic neurons in m3 are positive for Pax6, similar to the glutamatergic neurons produced in m4v. Thus, in addition to the putative Gata2–Tal2 complex, the two factors may also have functions independent of each other or their loss can be differentially compensated by other associating factors.

### Distinct Gata/Tal complexes regulate GABAergic neurons in different spatio-temporal patterns?

Gata2/3 and Tal1/2 also mark a subset of differentiating GABAergic neurons in the r1. Ventrally this GABAergic subdomain is bordered by Nkx2.2 positive serotonergic region and dorsally it extends to the dorsal boundary of *Nkx6.1* expression (Achim et al., 2012). However, requirement for the Gata and Tal factors in the r1 is different from the midbrain. In contrast to the midbrain, Tal1 is required for GABAergic differentiation in the rhombomere 1 (Achim et al., 2012). Similar to Tal2 in the midbrain, Tal1 may perform the selector function in a complex together with Gata factors. It is likely that this variation is used for generating GABAergic neuron subpopulations, which differ in their gene expression patterns and cellular phenotypes.

As discussed above, the Gata and Tal TFs may also operate independent of each other in distinct gene regulatory complexes. This is particularly evident in the r1, where *Gata2* and *Gata3* are strongly expressed in the developing serotonergic neurons without any detectable Tal1 or Tal2. Therefore, the TF complex regulating serotonergic neuron differentiation must differ from the one regulating the GABAergic phenotype. It will be of interest to analyse whether Gata2 and Gata3 also perform a terminal selector gene function in the serotonergic neuron lineage. If this is the case, Tal1, Tal2 and additional Gata cofactors may differentiate between serotonergic and GABAergic lineages.

## Conclusions

Here we demonstrate distinct requirements for the putative Gata cofactors Tal2 and Tal1 in differentiating GABAergic neurons in the midbrain. We suggest that variants of Gata–Tal complex function as terminal selectors identifying different types of post-mitotic GABAergic precursors. Although Gata2 and Tal2/Tal1 likely co-operate as GABAergic neuron determinants, differences in the midbrain neuronal differentiation in the absence of Gata2 or Tal1/2 function also suggest that these factors have unique targets. Knowledge on developmental regulation of the subgroups of GABAergic neurons in the midbrain–r1 region will allow later studies of their molecular composition, morphology and function.

## Supplementary Material

Supplementary Material
