# Supplementary material for: The role of Tal2 and Tal1 in the differentiation of midbrain GABAergic neuron precursors
Source: Biol Open. 2013 Aug 9;2(10):990–7. doi: 10.1242/bio.20135041 (PMC3798194; doi:10.1242/bio.20135041)
Supplement: Supplementary Material [file supp_2_10_990__index.html]

The role of Tal2 and Tal1 in the differentiation of midbrain GABAergic neuron precursors — The role of Tal2 and Tal1 in the differentiation of midbrain GABAergic neuron precursors — Supplementary Material 

# The role of *Tal2* and *Tal1* in the differentiation of midbrain GABAergic neuron precursors

## bio.20135041 Supplementary Material

**Files in this Data Supplement:**

- Supplementary Material - Kaia Achim et al. doi: 10.1242/bio.20135041
