# Supplementary material for: The role of Tal2 and Tal1 in the differentiation of midbrain GABAergic neuron precursors
Source: Biol Open. 2013 Aug 9;2(10):990–7. doi: 10.1242/bio.20135041 (PMC3798194; doi:10.1242/bio.20135041)
Supplement: Supplementary Material [file supp_bio.20135041_bio.20135041-s1.pdf]

Biology Open

Supplementary Material

Kaia Achim et al. doi: 10.1242/bio.20135041

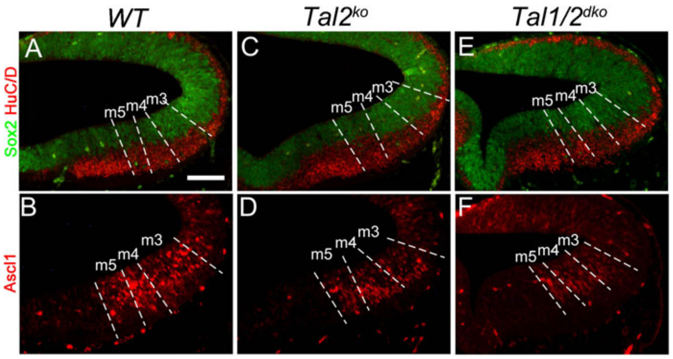

Fig. S1. Loss of *Tal2* or *Tal1/2* does not affect the gene expression in the ventricular zone. Immunostainings on E11.5 *WT* (**A**,**B**), *Tal2*^{ko} (**C**,**D**) and *Tal1/2*^{dko} (**E**,**F**) midbrains with anti-Sox and anti-HuC/D (A,C,E), and anti-Ascl1 (B,D,F). Dashed lines indicate the borders of midbrain m3–m5 domains, defined by the expression of *Nkx2.2* in the MZ (ISH on an adjacent section, not shown). Scale bar: 100 µm.

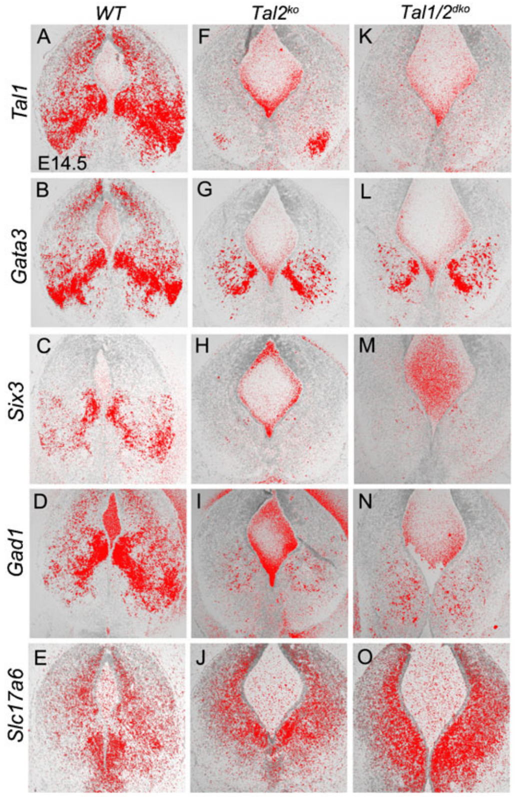

Fig. S2. Expression of GABAergic and glutamatergic marker genes in wild-type, *Tal2*^{ko} and *Tal1/2*^{dko} mutant midbrain at E14.5.
(**A–O**) Radioactive ISH on coronal parallel frozen sections of the midbrain, with the probes indicated. Few residual cells still express *Gad1* in mutants, whereas the expression of glutamatergic marker *Slc17a6* is strongly upregulated.

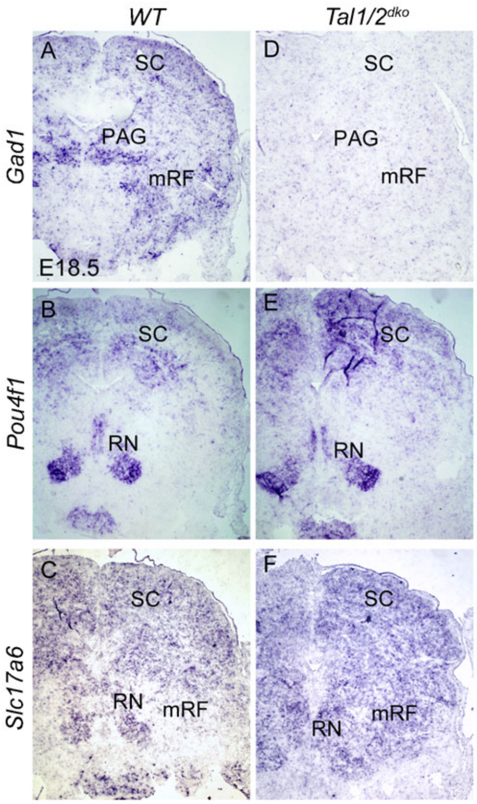

Fig. S3. Expression of *Gad1*, *Pou4f1*, and *Scl17a6* in wild-type and *Tal1/2*^{dko} midbrains at E18.5. (**A–F**) ISH on parallel coronal frozen sections of the midbrain with the probes indicated. SC, superior colliculi, PAG, periaqueductal grey, mRF, midbrain reticular formation, RN, red nucleus.
